# Supplementary material for: Effects of Re, W and Co on dislocation nucleation at the crack tip in the γ-phase of Ni-based single-crystal superalloys by atomistic simulation
Source: R Soc Open Sci. 2019 Jul 24;6(7):190441. doi: 10.1098/rsos.190441 (PMC6689646; doi:10.1098/rsos.190441)
Supplement: Supplementary materials for: Effects of Re, W, and Co on dislocation nucleation at the crack tip in the γ-phase of Ni-based single-crystal superalloys by atomistic simulation [file rsos190441supp1.pdf]

Supplementary materials for: Effects of Re, W, and Co on dislocation nucleation at the crack tip in the γ-phase of Ni-based single-crystal superalloys by atomistic simulation

Dianwu Wang[1], Chongyu Wang[1,2], and Tao Yu[1]

1.Central Iron and Steel Research Institute, Beijing 100081, China.
2.Department of Physics, Tsinghua University, Beijing 100084, China.
Corresponding author: Chongyu Wang.cywang@mail.tsinghua.edu.cn

The test of the model size along the crack front in the pure Ni system under the condition $K_I = 0.64K_{Ic}$ is given in Figure S1. The ΔE_{act} grows almost linearly with the crack front length. This result is reasonable as the saddle state dislocation spans the whole crack front length and the dislocation nucleation is expected to be more difficult with the increase of the dislocation line length. The crack front in our study has a length of ∼72 $\AA$ along the crack front direction, which is sufficient to obtain an accurate activation energy of an isolated dislocation.

The simulations for each concentration of randomly doped alloying element are repeated four times and the ΔE_{act} are calculated from the average value of the simulated results. Table S1, Table S2, Table S3 and Table S4 list the three repeted simulations results for each concentration of randomly doped alloying element. The values of interatomic energies are listed in Table S5.

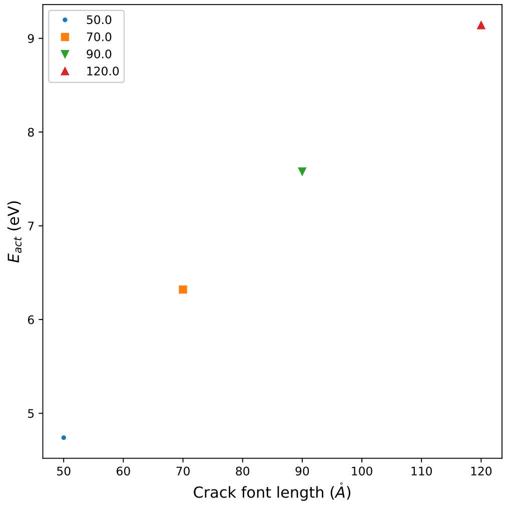

Figure S1: The variation of ΔE_{act} with the crack front length.

Table S1: Activation energy of dislocation nucleation ΔE_{act} (eV) at different loads.

System	$0.64K_{Ic}$	$0.68K_{Ic}$	$0.72K_{Ic}$	$0.76K_{Ic}$	$0.80K_{Ic}$
Ni	6.32	4.15	2.34	1.22	0.53
1$at.$%Re	5.89	3.67	1.93	0.94	0.34
2$at.$%Re	5.29	3.30	1.70	0.68	0.19
1$at.$%W	5.64	3.42	1.75	0.78	0.24
2$at.$%W	4.85	3.27	1.27	0.40	0.06
1$at.$%Co	6.18	4.01	2.27	1.18	0.52
2$at.$%Co	6.06	3.87	2.07	1.08	0.46

Table S2: Activation energy of dislocation nucleation ΔE_{act} (eV) at different loads.

System	$0.64K_{Ic}$	$0.68K_{Ic}$	$0.72K_{Ic}$	$0.76K_{Ic}$	$0.80K_{Ic}$
Ni	6.32	4.15	2.34	1.22	0.53
1$at.$%Re	6.18	4.00	2.21	1.15	0.41
2$at.$%Re	5.22	3.16	1.63	0.66	0.18
1$at.$%W	6.15	3.84	2.13	1.09	0.37
2$at.$%W	4.44	2.70	1.24	0.43	0.04
1$at.$%Co	6.16	4.00	2.22	1.16	0.51
2$at.$%Co	6.03	3.85	2.17	1.15	0.47

Table S3: Activation energy of dislocation nucleation ΔE_{act} (eV) at different loads (dash line denotes no energy barrier).

System	$0.64K_{Ic}$	$0.68K_{Ic}$	$0.72K_{Ic}$	$0.76K_{Ic}$	$0.80K_{Ic}$
Ni	6.32	4.15	2.34	1.22	0.53
1$at.$%Re	6.05	3.82	2.17	1.11	0.46
2$at.$%Re	4.90	2.78	1.36	0.40	–
1$at.$%W	5.89	3.66	1.96	1.05	0.43
2$at.$%W	4.06	2.05	0.87	0.13	–
1$at.$%Co	6.20	4.06	2.25	1.14	0.46
2$at.$%Co	5.79	3.61	2.10	1.04	0.41

Table S4: Activation energy of dislocation nucleation ΔE_{act} (eV) at different loads (dash line denotes no energy barrier).

System	$0.64K_{Ic}$	$0.68K_{Ic}$	$0.72K_{Ic}$	$0.76K_{Ic}$	$0.80K_{Ic}$
Ni	6.32	4.15	2.34	1.22	0.53
1$at.$%Re	5.78	3.63	1.98	0.94	0.35
2$at.$%Re	5.06	2.86	1.57	0.66	0.11
1$at.$%W	5.54	3.42	1.86	0.84	0.30
2$at.$%W	4.19	2.30	1.13	0.36	–
1$at.$%Co	5.99	3.83	2.09	1.09	0.46
2$at.$%Co	6.20	3.99	2.23	1.18	0.51

Table S5: Interatomic energy (units in eV) for alloying atom and Ni atom pair across the inclined (111) slip plane. The models are named with subplot labels (a)–(f) in Figure 11 of the article.

State	System	X-Ni(L1)	X-Ni(L3)	System	X-Ni(U4)	X-Ni(U6)
Initial	Model(a),X=Ni	-0.12	-0.16	Model(c),X=Ni	-0.13	-0.13
Saddle	Model(d),X=Ni	-0.13	-0.08	Model(f),X=Ni	-0.12	-0.09
Initial	Model(a),X=Re	-0.73	-0.60	Model(c),X=Re	-0.64	-0.67
Saddle	Model(d),X=Re	-0.81	-0.31	Model(f),X=Re	-0.76	-0.41
Initial	Model(a),X=W	-0.85	-0.74	Model(c),X=W	-0.78	-0.80
Saddle	Model(d),X=W	-0.97	-0.37	Model(f),X=W	-0.91	-0.49
Initial	Model(b),X=Co	-0.30	-0.24	Model(c),X=Co	-0.26	-0.27
Saddle	Model(e),X=Co	-0.30	-0.10	Model(f),X=Co	-0.29	-0.12
